# Microbial Urease in Health and Disease

**DOI:** 10.1371/journal.ppat.1004472

**Published:** 2014-12-11

**Authors:** Diego Mora, Stefania Arioli

**Affiliations:** Department of Food Environmental and Nutritional Sciences, University of Milan, Milan, Italy; University of North Carolina at Chapel Hill School of Medicine, United States of America

## Overview

Since the discovery of *Helicobacter pylori*, the urease activity of this bacterial pathogen has been identified as the key factor in infection and acid acclimation in the human stomach. Ureolytic activity plays a key role in the pathogenesis of several bacteria, and urease has also been described as an emerging pathogenic factor during fungal infection. However, urease produced by the oral bacteria community has been shown to counteract caries, and caries-free subjects have high levels of urease activity in plaque samples. Some lactic acid bacteria with documented probiotic behavior are urease-positive. Likewise, other lactic acid bacterial species that are widely used in yogurt production and other fermented dairy products use urease activity to counteract acid stress and to feed several biosynthetic pathways with carbon dioxide and ammonia derived from urea hydrolysis. Urease is also diffused in several species belonging to the human gut microbiota, and it is estimated that this complex microbial community is able to hydrolyze 15%–30% of the urea synthesized in normal subjects. In this context, urease was proposed to serve as a microbial biomarker to distinguish microbiomes based on age and geography, thus highlighting the crucial involvement of this enzymatic activity in nitrogen recycling when dietary nitrogen is limiting. In light of these considerations, the designation of urease as a microbial virulence factor would be misleading, and the proposed use of urease as a therapeutic target to counteract microbial infections should be carefully evaluated.

## Urease: Multifunctional Roles in Microbial Physiology

**Figure 1:**
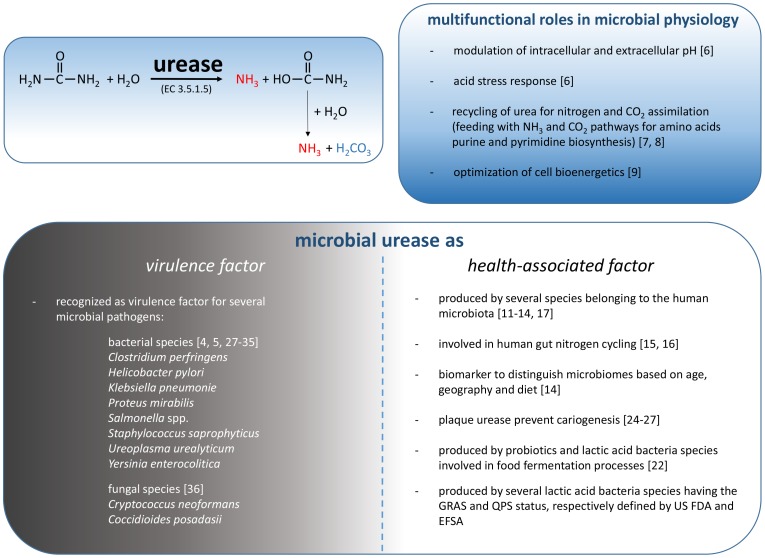
Schematic representation of the reaction catalyzed by microbial urease and the involvement of these enzymes in microbial physiology, human health, and disease.

Urease and its substrate urea represent historically important milestones in early scientific investigation. Urea was the first organic molecule synthesized, and urease from jack bean was the first enzyme crystallized, in addition to being the first enzyme shown to contain nickel [1]–[4]. The scientific interest in microbial urease is largely related to the relevance of this enzymatic activity in infection. This interest has been strongly stimulated since the discovery of the association of *H. pylori* with gastritis and stomach cancer [5]. Moreover, urease has served as a paradigm for understanding the activation mechanisms of many metalloenzymes that require accessory proteins for their catalytic activity [3], [4]. Urease is a urea amidohydrolase (EC 3.5.1.5) that catalyzes the hydrolysis of urea to yield ammonia and carbamate, which spontaneously decomposes to yield a second molecule of ammonia and carbonic acid. The released carbonic acid and the two molecules of ammonia are in equilibrium with their deprotonated and protonated forms, respectively, and the net effect of these reactions is an increase in the pH of the environment that surrounds the urease-positive microorganisms (Fig. 1). For this reason, urease is considered a stress response that was developed by several bacteria to counteract a low environmental pH [6]. In *Streptococcus thermophilus*, urease is metabolically related to the biosynthetic pathways involved in aspartate, glutamine, arginine, and carbon dioxide metabolism [7], [8]. Notably, urea hydrolysis increases the catabolic efficiency of *S. thermophilus* by modulating the intracellular pH and increasing the activity of β-galactosidase, glycolytic enzymes, and lactate dehydrogenase [9]. Urea hydrolysis results in increases in both the pH_in_ and the pH_out_ due to the rapid diffusion of ammonia outside of the cell. Consequently, in the presence of urea and a urease-positive microorganism, urease-negative microorganisms share the environmental benefit derived from the transient local pH increase [9].

Beyond that, the role of urease in most of the microorganisms that show this enzymatic activity is primarily linked to the recycling of nitrogenous wastes and nitrogen assimilation.

## Human Microbiota Urease As a “Health-Associated Factor”

Urea is the major nitrogenous waste product of most terrestrial animals. Ammonium, released from the urea present in the secretions of major and minor exocrine glands, provides a nitrogen source for bacteria that colonize the human body. It was estimated that 15%–30% of the urea synthesized in healthy subjects is continually hydrolyzed by microbial ureases [10]. Several microbial species belonging to the human microbiota produce active urease, and these species take advantage of urea hydrolysis, as has been demonstrated for the oral bacteria *Streptococcus salivarius* [11] and *Actinomyces naeslundii* [12] and hypothesized for other species of the gut microbiota [13], [14]. Urea is also present in human milk. Human milk contains only approximately 15% of its nitrogen in the form of urea. It is therefore believed that the total nitrogen in the infant lower gastrointestinal tract (GIT) may be present at suboptimal levels [15]. An increase in postnatal nitrogen levels is likely necessary to satisfy the growth and metabolism requirements of the infant and the GIT microbiota. In human infants, it has been determined that the amino acids in plasma can be derived from urea after hydrolysis and utilization of nitrogen by the intestinal microbiota [16]. It is therefore not surprising that an early colonizer of the GIT of humans, the *Bifidobacterium longum* subsp. *Infantis*, produced an active urease [17]. By contrast, in subjects with acute liver failure, the major clinical problem is the development of hepatic encephalopathy (HE) that is associated with high level of gut-derived ammonia. In this scenario, the microbial gut community, especially urease-positive species such as *Klebsiella* spp. and *Proteus* spp., is an important source of ammonia in humans in the pathogenesis of HE [18]. Several clinical cases have suggested that the severity of HE can be reduced by modulating the microbial gut community using agents that lead to a normalization of gut microbiota, such as rifaximin, lactulose, prebiotics, and probiotics. However, even probiotics can be urease-positive. Interestingly, a recent study performed using a murine model reported that the administration of the probiotic urease-positive *Lactobacillus reuteri* reduced the amount of urease activity in the murine gut, presumably due to the suppression of fecal bacteria [19]. Another bacterial species that is currently used as a probiotic for the oropharyngeal tract is the urease-positive *S. salivarius* strain K12 [20]. Following oral administration, strain K12 can colonize the oral mucosae of infants and adults and down-regulate the innate immune responses of human epithelial cells. It is also active against *S. pyogenes* and safe and well tolerated by the human host [21]. In a more general food context, it is worth mentioning that yogurt consumption is commonly associated with a health benefit by the consumers [21], and one of the two species of the yogurt consortium, *S. thermophilus*, is urease-positive [22]. *S. thermophilus* is widely used in the manufacturing of dairy products, yogurt, fermented milk, and cheeses, and as a consequence, over 10^21^ living cells carrying active urease molecules are ingested annually by the human population.

A recent study [14] focused on the characterization of gut microbial communities in two human populations revealed that urease gene frequency was significantly higher in Malawian and Amerindian infant microbiomes and that it decreased with age in these two populations, unlike in the United States, where it remains low from infancy to adulthood. Considering that urease has a crucial involvement in nitrogen recycling, particularly when diets are deficient in protein, the ability of the microbiome to use urea would presumably be advantageous to both microbes and host.

Urea is secreted into all parts of the digestive tract starting from the oral cavity. In saliva, urea is present at a concentration of 3–10 mM, and it represents a relevant nitrogen source for several species belonging to the oral microbiota, including *S. salivarius*, *S. vestibularis* and *Actinomyces naeslundii*. A substantial body of evidence is beginning to accumulate that indicates a direct contribution of alkali generation in dental biofilms to the inhibition of dental caries [23]. The development of dental caries is favored by tooth demineralization that happens as a consequence of the frequent acidification of dental biofilms and the subsequent emergence of acidogenic and acid-tolerant microorganisms, including mutans streptococci and *Lactobacillus* spp., which ferment dietary carbohydrates rapidly and lower the pH. The increasing number of acid-tolerant microorganisms results in a simultaneous decrease in the less acid-tolerant species that are often associated with dental health [24]. Notably, bacteria associated with dental health are able to use urea and/or arginine to generate ammonia. Alkali production by these microorganisms positively affects the balance between the remineralization and demineralization of the tooth and may help prevent the emergence of cariogenic microorganisms [25]. The real scenario is actually more complex than it might appear. In fact, while the urease activity associated with plaque seems to correlate with a decrease in the incidence of caries, the urease activity associated with the saliva had a significant effect on the risk of developing caries, and this effect was not protective but instead promoted the development of caries [26]. It therefore appears that the oral localization of urease activity is fundamental in preventing caries. Interestingly, in mice, the carcinogenicity of the plaque bacterium *S. mutans* (naturally urease-negative) was dramatically reduced in a derivative recombinant strain of *S. mutans* that was able to produce an active urease, thus suggesting that recombinant ureolytic bacteria may be useful in promoting dental health [27].

## Microbial Urease As a General “Virulence Factor”

In addition to the positive aspects of microbial ureases in human health, a consistent body of evidence has identified urease as a virulence factor for several microbial pathogens (Fig. 1). In fact, ureolytic activity has a key role in the pathogenesis of bacteria such as *Clostridium perfringens*, *Helicobacter pylori*, *Klebsiella pneumoniae*, *Proteus mirabilis*, *Salmonella* spp., *Staphylococcus saprophyticus*, *Ureoplasma urealyticum*, and *Yersinia enterocolitica*, and such activity has been reported in diseases such as urolithiasis, pyelonephritis, ammonia encephalopathy, HE, hepatic coma, and gastroduodenal infections [4], [28]. The role of urease in microbial infection has been well established in *H. pylori*. Hydrolysis of urea in the human stomach provides NH_3_ that is essential for acid neutralization, enabling *H. pylori* to raise the pH in its microenvironment and periplasm, thus maintaining the proton motive force [28]. Moreover, the urea-dependent ammonia production appears to be partially responsible for the gastric mucosal injury found in association with *H. pylori* infection [29]. A proton-gated channel, UreI, which regulates the uptake of urea [4], is only active at acidic pH and therefore does not allow for the transport of urea into the bacterial cell at neutral pH, thus preventing lethal alkalinization of the cytoplasm [4]. Without this mechanism, *H. pylori* is unable to develop the infection process in the stomach [30], [31]. Similarly, the urease activity allows the survival to the gastric transit of *Y. enterocolitica* [32].

The role of urease activity in urinary tract infections and struvite and carbonate apatite stones formation was described for *P. mirabilis* and *Sta. saprophyticus*. The urease-dependent invasive property of *P. mirabilis* was supported by in vitro observation and by the use of urease-negative mutants. *P. mirabilis* defective in urease exhibited in a mouse model an ID50 more than 1,000-fold higher than the wild-type strain, and only the wild-type strain was able to persist significantly [33]. Likewise, the contribution of urease to the cytopathogenicity of *Sta. saprophyticus* has been demonstrated in a rat model using a chemically mutagenized urease-deficient strain, and by the heterologous expression of an active urease in the nonureolytic *Staphylococcus carnosus* strain [34], [35].

More recently [36], the role of urease as a general microbial virulence factor was proposed, highlighting the emerging pathogenic roles of urease during infection of the fungal species *Cryptococcus neoformans* (a basidiomycete) and *Coccidioides posadasii* (an ascomycete). During fungal lung infection, the urea present in the epithelial lining fluid of the lungs is hydrolyzed by fungal urease, and the generated ammonia inhibits immune function and contributes to lung tissue damage [36].

Other human pathogens are urease-positive, and in many cases, urea hydrolysis is thought to have a role in the infectivity or persistence of the microorganisms. In this context, although largely unexplored, the positive role of urease in microbial physiology (Fig. 1) can be an advantage for a pathogen during the various stages of the infection process in terms of competition with commensal microorganisms associated with the human body.

## Perspectives

Because of the facts that the human genome does not contain urease-encoding genes and that no human nickel-containing enzymes are known, urease was proposed as a potential therapeutic target [36] without taking into consideration all the positive aspects linked to the microbial ureases of the human microbiota. In this context, the use of the term “virulence factor” for microbial ureases should be carefully evaluated. Microbiologists working on infectious organisms routinely define any gene product that contribute to the virulence potential of a pathogen as a “virulence factor.” Recently, the increasing interest in the human microbiota raises questions about the terminology we use to describe the molecular and metabolic strategies that pathogenic microbes use to compete in these complex biological systems [37]. In the GIT, many pathogens and commensals use similar strategies to overcome the challenges associated with this particular environment. It would therefore be misleading to describe the same strategies and structures found in harmless or beneficial commensals as “virulence factors” simply because they were acquired or evolved to survive in the GIT. The term “niche factors” was therefore proposed [37] to describe the molecular and metabolic strategies evolved by beneficial gut microbes to colonize this complex environment.
